# Enhancing Giardicidal Activity and Aqueous Solubility through the Development of “RetroABZ”, a Regioisomer of Albendazole: In Vitro, In Vivo, and In Silico Studies †

**DOI:** 10.3390/ijms241914949

**Published:** 2023-10-06

**Authors:** Carlos Martínez-Conde, Blanca Colín-Lozano, Abraham Gutiérrez-Hernández, Emanuel Hernández-Núñez, Lilián Yépez-Mulia, Luis Fernando Colorado-Pablo, Rodrigo Aguayo-Ortiz, Jaime Escalante, Julio C. Rivera-Leyva, Jessica Nayelli Sánchez-Carranza, Elizabeth Barbosa-Cabrera, Gabriel Navarrete-Vazquez

**Affiliations:** 1 Facultad de Farmacia, Universidad Autónoma del Estado de Morelos, Cuernavaca 62209, Morelos, Mexico; mcc_ff@uaem.mx (C.M.-C.); clbi_ff@uaem.mx (B.C.-L.); ghaa_ff@uaem.mx (A.G.-H.); julio.rivera@uaem.mx (J.C.R.-L.); jessica.sanchez@uaem.mx (J.N.S.-C.); 2 Departamento de Recursos del Mar, Centro de Investigación y de Estudios Avanzados, IPN, Unidad Mérida, Merida 97310, Yucatán, Mexico; emanuel.hernandez@cinvestav.mx; 3 Unidad de Investigación Medica en Enfermedades Infecciosas y Parasitarias, Unidad Médica de Alta Especialidad-Hospital de Pediatría, Centro Médico Nacional Siglo XXI, Instituto Mexicano del Seguro Social, Mexico City 06720, Mexico; lilianyepez@yahoo.com; 4 Departamento de Farmacia, Facultad de Química, Universidad Nacional Autónoma de Mexico, Mexico City 04510, Mexico; fernando1303@comunidad.unam.mx (L.F.C.-P.); rodaguayo@comunidad.unam.mx (R.A.-O.); 5 Centro de Investigaciones Químicas-IICBA, Universidad Autónoma del Estado de Morelos, Av. Universidad 1001, Cuernavaca 62209, Morelos, Mexico; jaime@uaem.mx; 6 Sección de Estudios de Posgrado e Investigación, Escuela Superior de Medicina, IPN, Mexico City 11340, Mexico; rebc78@yahoo.com.mx

**Keywords:** benzimidazole, giardicidal, albendazole, solubility, docking

## Abstract

Parasitic diseases, including giardiasis caused by *Giardia lamblia* (*G. lamblia*), present a considerable global health burden. The limited effectiveness and adverse effects of current treatment options underscore the necessity for novel therapeutic compounds. In this study, we employed a rational design strategy to synthesize retroalbendazole (**RetroABZ**), aiming to address the limitations associated with albendazole, a commonly used drug for giardiasis treatment. **RetroABZ** exhibited enhanced in vitro activity against *G. lamblia* trophozoites, demonstrating nanomolar potency (IC_50_ = 83 nM), outperforming albendazole (189 nM). Moreover, our in vivo murine model of giardiasis displayed a strong correlation, supporting the efficacy of **RetroABZ**, which exhibited an eleven-fold increase in potency compared to albendazole, with median effective dose (ED_50_) values of 5 µg/kg and 55 µg/kg, respectively. A notable finding was **RetroABZ**’s significantly improved water solubility (245.74 µg/mL), representing a 23-fold increase compared to albendazole, thereby offering potential opportunities for developing derivatives that effectively target invasive parasites. The molecular docking study revealed that **RetroABZ** displays an interaction profile with tubulin similar to albendazole, forming hydrogen bonds with Glu198 and Cys236 of the β-tubulin. Additionally, molecular dynamics studies demonstrated that **RetroABZ** has a greater number of hydrophobic interactions with the binding site in the β-tubulin, due to the orientation of the propylthio substituent. Consequently, **RetroABZ** exhibited a higher affinity compared to albendazole. Overall, our findings underscore **RetroABZ**’s potential as a promising therapeutic candidate not only for giardiasis but also for other parasitic diseases.

## 1. Introduction

**Figure 1:**
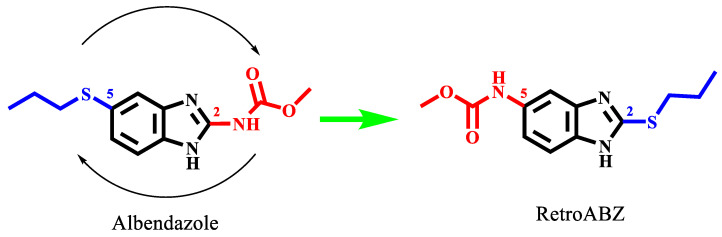
Design of albendazole regioisomer: **RetroABZ**.

Parasitic diseases have emerged as a significant worldwide public health concern, affecting millions of individuals [1]. These diseases are being increasingly favored by various factors, including environmental and climate changes, overpopulation, and globalization [2]. Notably, certain parasitic diseases caused by protozoa fall under the category of neglected tropical diseases, often closely associated with poor hygiene and sanitation conditions [3]. Among these diseases, giardiasis, caused by *G. lamblia* (also known as *Giardia duodenalis* or *Giardia intestinalis*), was classified as a neglected tropical disease in 2004 [4]. Giardiasis stands as the most prevalent protozoan infection in humans, with more than 200 million people worldwide [5], with 50% to 75% of cases resulting from asymptomatic infection [6]. Chronic *Giardia* infections predominantly affect pediatric populations and are linked to persistent diarrhea, growth failure, and cognitive function alterations [5,7]. Currently, several drugs are available for treating symptomatic giardiasis, primarily belonging to the nitroimidazole class, such as metronidazole, tinidazole, secnidazole, and nitazoxanide of the nitrothiazole chemotype [8,9]. However, their clinical utility is limited due to side effects and the emergence of clinical resistance [10]. Alternatively, benzimidazole compounds, namely albendazole and mebendazole, have been reported for their potential in *Giardia* treatment. The compounds of interest, benzimidazole derivatives, demonstrate a lower toxicity profile compared to nitroimidazole-type compounds, making them potential alternatives for helminth and protozoan treatment [11]. Previous reports have supported the therapeutic potential of benzimidazole derivatives in this regard [8,9,12,13]. However, recent investigations have reported resistance of *G. lamblia* to benzimidazole-2-carbamate compounds associated with mutations in β-tubulin E198K [14]. In addition to the above, these compounds suffer from limited water solubility. For instance, albendazole, with a reported solubility of 0.2 µg/mL, exhibits poor gastrointestinal tract absorption, resulting in an oral bioavailability of merely 5%, thereby restricting its use in treating systemic infections [15,16]. The emergence of drug resistance poses a significant obstacle in mitigating the effects of *G. lamblia* infection. Furthermore, the hindered oral bioavailability resulting from its low water solubility exacerbates this issue. Hence, there is an imperative to devise novel compounds with promising antiparasitic properties. In this study, we address these limitations by reporting the design and synthesis of a novel regioisomer compound named **RetroABZ** that involves modifying the position of the side groups of albendazole (Figure 1).

**Figure 2:**
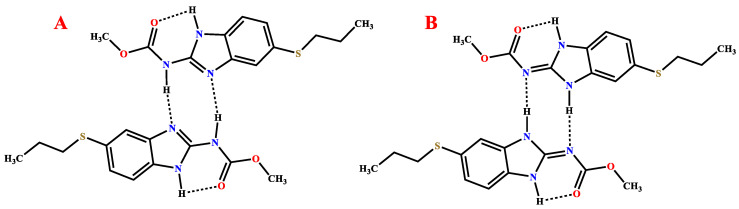
Dimeric structure of desmotropes (**A**) albendazole I and (**B**) albendazole II, adapted from ref. [17].

**RetroABZ** is an outcome of a well-thought-out rational design approach that addresses the limitations of current compounds used for treating parasitosis. Albendazole, a broad-spectrum active ingredient effective against parasitic infestations in both humans and animals, suffers from significant drawbacks such as poor solubility and low oral bioavailability. The limited water solubility of benzimidazole carbamates is attributed to the existence of intermolecular hydrogen bonds. Recent research indicates that albendazole’s desmotropy arises from its polymorphic nature, wherein two distinct dimeric conformations, namely forms I and II, coexist [17] (Figure 2).

The presence of intermolecular hydrogen bonding in these dimers is a notable feature, and it is believed that these dimeric networks play a role in the low aqueous solubility of albendazole. To address this limitation, in the present study, the development of a regioisomer of albendazole was pursued, involving modifications to the position of the side groups of the benzimidazole core, with the aim to disrupt the formation of dimeric conformations and consequently enhance the solubility of the new compound named **RetroABZ**, while maintaining or possibly increasing its antiparasitic activity. Herein, we present the collection of in vitro antiprotozoal evaluations combined with in silico and in vivo estimations leading to the concept of in combo screening in drug discovery [18]. This research reflects their dedicated endeavor towards rational drug design with the ultimate goal of enhancing therapeutic agents. Antiparasitic drugs are essential for controlling infections stemming from a range of protozoa, helminths, and ectoparasites. The choice of treatment is dependent upon the particular causative agent within each category. While there exists a repertoire of available drugs, they are not without significant drawbacks, including issues of low solubility and limited bioavailability. Additionally, the emergence of drug resistance poses a substantial concern.

## 2. Results and Discussion

### 2.1. Drug Design and Chemical Synthesis

In this study, a novel compound, **RetroABZ**, was designed and synthesized as an albendazole regioisomer and evaluated for its potential as a giardicidal agent. The key characteristic of **RetroABZ** is the exchange of side groups with albendazole, aiming to enhance its biopharmaceutical properties, particularly solubility, while maintaining or possibly increasing its antiparasitic activity. Prior studies have indicated that the poor aqueous solubility of benzimidazole-2-carbamates may result from intramolecular and intermolecular hydrogen bonding, leading to dimeric arrangements known as desmotropes of albendazole [17]. Desmotropy, an infrequent phenomenon associated with tautomerism, involves both tautomeric forms being isolable in the solid state. With the regioisomer design, **RetroABZ**, involving the interchange of substituents at positions 2 and 5 of the benzimidazole core, the potential disruption of intramolecular and intermolecular hydrogen bonds may lead to the loss of the crystalline structure, thereby improving its biopharmaceutical properties. Compound **1** (**RetroABZ**) was obtained in four steps, with good yield and reasonable reaction time. The spectroscopic and spectrometric studies were consistent with the expected structure.

### 2.2. In Vitro Activity against Giardia lamblia

The bioassay results against *G. lamblia* are presented in Table 1. **RetroABZ** demonstrated a giardicidal potency in the nanomolar range with a median inhibitory concentration (IC_50_) of 83 nM, meanwhile albendazole displayed an IC_50_ value of 196 nM. **RetroABZ** exhibited higher giardicidal activity than albendazole, being 2.4 times more active than albendazole.

### 2.3. Cytotoxicity in Human Hepatocellular Carcinoma Cell Line and Immortalized Human Keratinocytes

**Figure 3:**
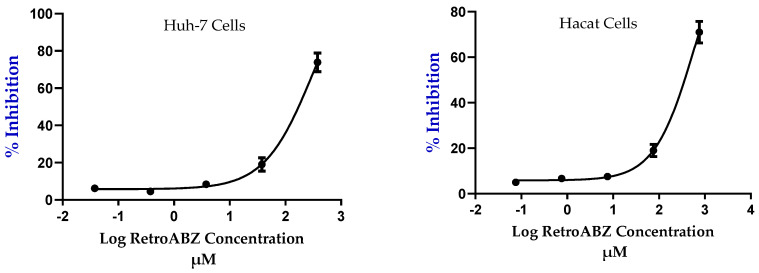
Cytotoxicity of **RetroABZ** in Huh−7 hepatic cells and HaCaT keratinocytes.

The cytotoxicity of **RetroABZ** against a human hepatocellular carcinoma cell line (Huh-7) and immortalized human keratinocytes (HaCaT) was evaluated (Table 1), revealing a very low median cytotoxic concentration (CC_50_ > 300 µM). The selection of Huh-7 cells in toxicological analyses stems from their metabolic profiles, which closely parallel those of typical hepatocytes. This metabolic resemblance is pivotal for accurate assessments of potential toxic biotransformation. The selectivity index (SI), calculated as the ratio of cytotoxicity to biological activity (SI = CC_50_ cells/IC_50_ parasites), is an indicator of whether the antiprotozoal activity of a compound is primarily due to its intrinsic cytotoxicity [19]. When the SI exceeds 10, it suggests that the antiprotozoal activity is not driven by the compound’s cytotoxicity [20]. In the case of **RetroABZ**, its nanomolar giardicidal activity combined with its very low cytotoxic effect in both cell lines (Figure 3) resulted in a significantly high selectivity index, surpassing 3500. Comparatively, it has been reported that albendazole exhibits a cytotoxic concentration (CC_50_) of 79.71 µM against Huh-7 and 118.2 µM vs. HaCaT [21]. These data imply that both **RetroABZ** and albendazole are much more selective against *G. lamblia* than against any mammalian cell.

### 2.4. Giardicidal Activity of **RetroABZ** in a Murine Model of Giardiasis

The antiprotozoal activity of the compound **RetroABZ** was evaluated in CD-1 mice infected with *G. lamblia* trophozoites following the methodology previously reported [20]. Table 2 presents the ED_50_ values for the compounds assessed. **RetroABZ** exhibited remarkably higher potent in vivo activity against *G. lambia*, with an ED_50_ value of 5 μg/kg (18.84 nmol/kg) of body weight in comparison to albendazole (55 μg/kg). **RetroABZ** demonstrated 11 times higher giardicidal activity than the reference drug, albendazole. Furthermore, **RetroABZ** exhibited 39-fold higher potency compared to metronidazole, which is another frequently prescribed drug for the treatment of giardiasis.

### 2.5. Solubility

**Figure 4:**
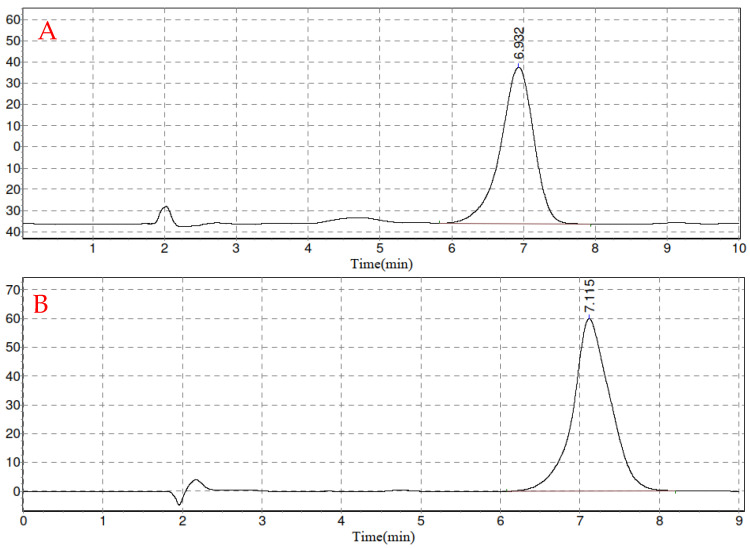
Retention time for **RetroABZ** (6.932 min, (**A**)) and albendazole (7.115 min, (**B**)).

Chromatographic conditions were optimized to achieve a short runtime, adequate retention, and acceptable peak shapes. Thus, different compositions of the organic diluents and the aqueous part (90:10, 80:20, 70:30, 60:40, *v*/*v*) were tested on a Phenomenex Luna C_18_ column. Finally, the most favorable separation for the analytes albendazole and **RetroABZ**, ensuring an adequate response and improved peak shape, was achieved using a mobile phase consisting of 60% methanol and 40% water (*v*/*v*) at a flow rate of 1 mL/min. Figure 4 illustrates the retention time for **RetroABZ** at 6.932 min, indicating a satisfactory outcome.

The water solubility of **RetroABZ** at 37 °C is presented in Table 3. It is noteworthy that the solubility of this new benzimidazole derivative was remarkably higher, measuring 245.7 μg/mL, in contrast to the experimental solubility of albendazole (10.55 μg/mL), reflecting a substantial 23-fold increase. It is remarkable to acknowledge that prior researchers have documented the solubility of albendazole at 25 °C to be 0.02 μg/mL [22].

**Figure 5:**
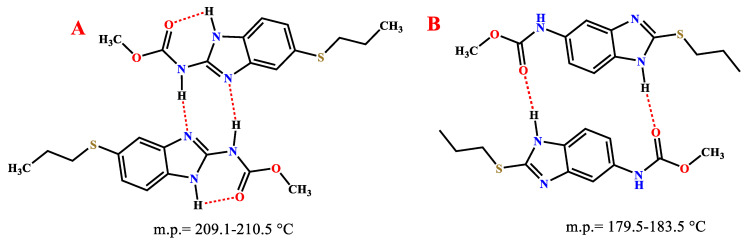
The potential disruption of inter– and intramolecular hydrogen bonds in the albendazole (**A**) and its regioisomer **RetroABZ** (**B**) may have an impact on their crystallinity, solubility, and melting point.

The substantial enhancement in **RetroABZ**’s solubility could be attributed to the disruption of the crystal lattice formed with hydrogen bonds in albendazole desmotropes, which are no longer present when the carbamate substituent shifts from position 2 to position 5. This observation is further supported with the observed decrease of 30 °C in **RetroABZ**’s melting point, indicating a weakened crystal lattice compared to albendazole, as depicted in Figure 5.

The efficacy of albendazole is frequently hindered by its limited intestinal absorption, primarily attributed to its low aqueous solubility. Enhancing its dissolution could potentially ameliorate this issue. This low water solubility leads to inadequate absorption from the gastrointestinal tract and consequently results in low oral bioavailability of albendazole (less than 5%), a substantial drawback for systemic use. Enhancing the aqueous solubility of small molecules is a pivotal approach to mitigate these challenges. One alternative strategy involves disrupting molecular planarity/symmetry [23]. The data obtained in the present study showcase a noteworthy enhancement in water solubility for **RetroABZ**, exhibiting a 23-fold increase when compared to albendazole. This advancement underscores the potential of **RetroABZ** as a significant improvement in this regard.

### 2.6. In Silico Pharmacokinetic Profile

The Lipinski rule of five has emerged as a significant parameter in rational drug development, yet its importance in the selection or discrimination of antiparasitic molecules has been subject to questioning. The strict application of these rules has led to the removal of numerous potential antiparasitic drugs from the priority list. Considering this aspect, an in silico pharmacological analysis was conducted to estimate the biopharmaceutical, pharmacodynamics, and pharmacokinetic profiles of the compounds. Previous studies have reported the use of an in silico pharmacological consensus analysis (PHACA), which combines biopharmaceutical, pharmacodynamic, pharmacokinetic, and toxic predictions to identify compounds with favorable properties [24]. For the estimation of the ADMET (absorption, distribution metabolism, excretion, and toxicity) profile of compounds **RetroABZ** and albendazole, the ADMETLab 2.0 (https://admetmesh.scbdd.com, accessed on 30 September 2022) predictor was utilized [25].

In the ADMET profile calculations (Table 4), **RetroABZ** exhibited substantial intestinal absorption prediction even better than that of albendazole, indicating a high likelihood of crossing the blood–brain barrier. The evaluation of distribution parameters, including plasma protein binding, ideally should be less than 95%, but interestingly, **RetroABZ** was the only compound that did not meet this criterion. Regarding metabolic stability, all compounds demonstrated low values as substrates for the primary metabolizing enzyme CYP3A4, while displaying higher values as substrates for the enzyme CYP2D6, which means that this enzyme will be the one that biotransforms the compound. Estimation of excretion parameters indicated satisfactory clearance values for both compounds, leading to a prolonged half-life of over 3 h. In terms of toxicity assessment, all compounds showed a low probability of hERG (human ether-a-go-go-related gene) channel blockade, indicating no cardiotoxicity. Furthermore, they exhibited low acute oral toxicity in rat models.

### 2.7. Docking Studies

**Figure 6:**
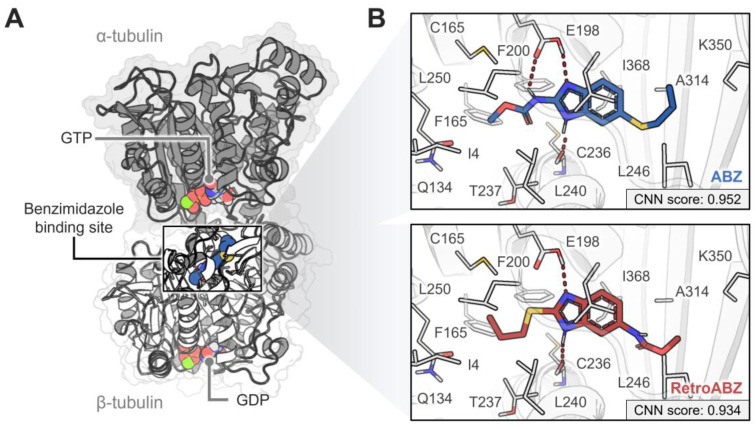
(**A**) Depiction of *G. lamblia* α/β-tubulin heterodimer model highlighting the binding sites of the GDP, GTP, and benzimidazoles (i.e., mebendazole, nocodazole, and parbendazole). (**B**) Binding mode of albendazole (upper panel) and **RetroABZ** (lower panel) at the benzimidazole-binding site.

After conducting in vitro antiparasitic assays, computational investigations were carried out to explore the potential molecular-level mechanisms of action of **RetroABZ**. In this work, we conducted the molecular modeling studies using the α/β-tubulin heterodimer of *G. lamblia*. This choice was guided by the established connection with the original compounds, as various benzimidazole carbamate compounds like albendazole have demonstrated their effectiveness against *Giardia* by inhibiting β-tubulin polymerization [26]. While the benzimidazole binding site is primarily located in β-tubulin, the inclusion of α-tubulin in our model is justified due to its interaction with the H8 helix of the β monomer [27]. This interaction is pivotal in influencing pocket formation and water exposure [28]. Moreover, it is crucial to consider the α subunit during the docking studies to ensure that the ligand predominantly binds within the pocket rather than at the protein’s interface. Figure 6 shows the outcomes of the molecular docking analysis, revealing that both **RetroABZ** and albendazole have the capability to bind within the same binding site in the β-tubulin as other carbendazim derivatives. The three-dimensional binding models for these compounds suggest the establishment of hydrogen bond (HB) interactions with the Glu198 side chain and Cys236 main chain. The CNN (convolutional neural networks) score demonstrated that both compounds exhibit similar affinities to the binding site.

### 2.8. Molecular Dynamics

**Figure 7:**
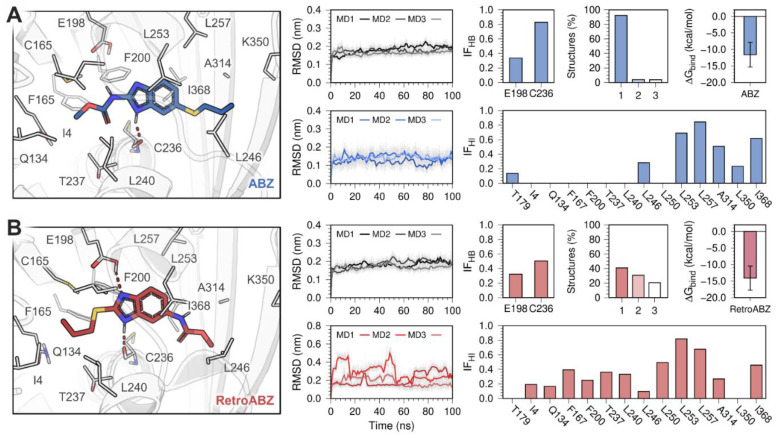
Analysis of the three independent 100 ns MD simulations of (**A**) albendazole and (**B**) **RetroABZ** complexed with *G. lamblia* α/β-tubulin model. On the left, the most representative binding mode extracted from the most populated cluster from the final 50 ns of the three MD simulations (MD1 to MD3). The graphs illustrate the RMSD of both the α/β-tubulin heterodimer backbone and the ligand throughout the 100 ns simulation. The plots on the right display the fraction of hydrophobic (IF_HI_) and hydrogen bond (IF_HB_) intermolecular interaction between the ligands and the protein, along with the percentage of structures occupying the first three clusters and the binding free energy computed via gmx_MMPBSA.

To assess the stability of protein–ligand complexes, we conducted three independent 100 ns molecular dynamics (MD) simulations using the GROMACS (Groningen Machine for Chemical Simulations) program. Figure 7 presents the analysis of the MD simulations for both systems. The RMSD (root-mean-square deviation) of the α/β-tubulin backbone demonstrated that the heterodimer reached equilibrium within the simulated time in all three replicates for both systems. On the other hand, the ligand RMSD suggests that albendazole exhibits greater stability at the binding site, displaying minor conformational changes within 0.2 nm. In contrast, **RetroABZ** shows a semi-stable state after 60 ns of simulations in all three replicates. An analysis of the hydrogen bond interaction fraction (IF_HB_) might indicate that the higher stability of albendazole compared to **RetroABZ** could be attributed to the former displaying hydrogen bonds with Glu198 and Cys236 for longer periods. This, in turn, led to a higher percentage of structures in the first cluster for albendazole. However, **RetroABZ** gains a clear advantage over albendazole by forming a greater number of hydrophobic interactions with the binding site. The benzimidazole site contains a deep region with numerous hydrophobic residues, typically accommodating the carbamate group. In the case of **RetroABZ**, the propylthio substituent is oriented towards this region, promoting interactions with the hydrophobic residues of this embedded pocket, and preserving the interactions formed with albendazole. Furthermore, the Molecular-Mechanics–Poisson–Boltzmann Surface Area (MM/PBSA) calculations demonstrated that these new hydrophobic interactions also resulted in a better affinity of **RetroABZ** (ΔG_bind_ = −14.09 ± 3.65 kcal/mol) compared to albendazole (ΔG_bind_ = −11.57 ± 3.76 kcal/mol). These findings suggest that these hydrophobic interactions could be responsible for the heightened antiparasitic effectiveness of **RetroABZ**. It is important to keep in mind that intermolecular interactions observed during molecular dynamics simulations should be interpreted with caution, as force fields typically involve estimation errors. In this case, the hydrogen bond formed between benzimidazole and the Glu198 residue may indeed have a longer average lifetime than estimated. Additionally, there might be other interactions like CH-π or π–π interactions that are not reliably analyzed using molecular mechanics approaches.

### 2.9. In Silico Pharmacological Consensus Analysis

To highlight the merits of the evaluated compound, an in silico pharmacological consensus analysis was conducted [24], taking into consideration the aforementioned results. As depicted in Table 5, **RetroABZ** displayed more favorable properties compared to albendazole, the reference drug used for treating giardiasis and the first-line agent for treating helminthic infections. **RetroABZ** exhibited 2.4 times greater in vitro activity against *G. lamblia* than albendazole, and the in vivo results exhibited a good correlation with the in vitro studies. It is worth noting that only a few adverse events related to albendazole treatment have been reported; however, most of these are associated with the use of high doses in systemic infections [29,30]. The notable improvement in the water solubility of **RetroABZ** creates opportunities for investigating new derivatives that can effectively target invasive parasites, addressing significant limitations associated with albendazole’s poor water solubility.

Several challenges might arise during further antiprotozoal drug development. It is crucial to refine lead compounds to align with specific product profiles, a step that frequently determines the pace of progress in drug discovery projects. Thorough evaluation in various animal models, with meticulous attention to formulation, will be essential. Notably, antiparasitic drug discovery and development do not primarily hinge on market forces. Therefore, it is imperative to engage the triple helix of academia, industry, and government, ensuring their collaborative involvement in the process.

## 3. Materials and Methods

### 3.1. Chemicals and Analytical Methods

The starting materials and solvents were purchased from Sigma-Aldrich (Burlington, MA, USA) and were utilized without any additional purification. Melting points were determined employing an EZ-Melt MPA120 automated melting point apparatus from Stanford Research Systems (Sunnyvale, CA, USA), and are uncorrected. Reactions were monitored using thin-layer chromatography on 0.2 mm precoated silica gel 60 F254 Merck plates (Darmstadt, Germany). ^1^H NMR spectra were recorded on a Varian Oxford (600 MHz) spectrometer (Palo Alto, CA, USA), while ^13^C NMR (150 MHz) spectra were acquired as well. Chemical shifts are expressed in ppm with respect to tetramethylsilane (Me_4_Si, δ = 0) in DMSO-d_6_ and CDCl_3_; *J* values are denoted in Hz. Abbreviations like *s* (singlet), *d* (doublet), *q* (quartet), *dd* (doublet of doublet), *t* (triplet), and *m* (multiplet) are used. Mass spectrometry data were obtained on a JEOL JMS-700 spectrometer (Tokyo, Japan) via electronic impact. Main spectra can be consulted in the Supplementary Material.

#### 3.1.1. Chemical Synthesis

**Scheme 1:**
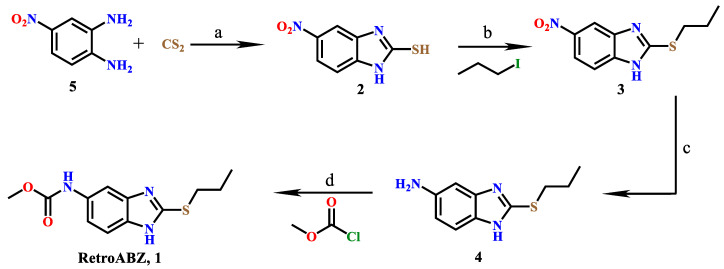
Reagent and conditions for the synthesis of **RetroABZ**: (**a**) EtOH, 2.5 equiv KOH, 80 °C, 12 h; (**b**) MeCN, K_2_CO_3,_ 2–4 °C, 8 h; (**c**) EtOH, 6 equiv Sn^0^, 7 equiv HCl, H_2_O, 80 °C, 8 h; (**d**) THF, NaHCO_3_, 2–4 °C, 8 h.

The sequence shown in Scheme 1 was followed. Compound **2** was prepared from 4-nitro-1,2-phenylenediamine (**5**) and a mixture of ethanol, potassium hydroxide, and carbon disulfide, to give 5-nitro-1*H*-benzo[d]imidazole-2-thiol (**2**). Subsequently, a mixture containing acetonitrile, potassium carbonate, and compound **2** was treated with propyl iodide, to afford 5-nitro-2-(propylthio)-1*H*-benzo[d]imidazole (**3**). The reduction of the nitro group was performed using a mixture of metallic tin, water, and hydrochloric acid at reflux, leading to 2-(propylthio)-1*H*-benzo[d]imidazol-5-amine (**4**). In the last step, amine **4** was reacted with methyl chloroformate in tetrahydofurane as a solvent at a low temperature to obtain the methyl carbamate **RetroABZ** (**1**).

#### 3.1.2. 5-Nitro-1*H*-benzo[d]imidazole-2-thiol (**2**)

The synthesis of compound **2** followed the method outlined by Van Allan [31]. Initially, a mixture of KOH (2.91 g, 52.2 mmol, 2 equiv) in ethanol was prepared, and CS_2_ (1.89 mL, 31.3 mmol, 1.2 equiv) was subsequently added. The resulting reaction mixture was stirred for 15 min at room temperature. Next, 4-nitrobenzene-1,2-diamine (4 g, 26.1 mmol) was added, and the mixture was further stirred at reflux for 12 h. After completion of the reaction, neutralization to pH 7 was achieved using a 10% hydrochloric acid solution. The solvent was then removed utilizing a high-vacuum system, and the resulting residue was suspended in cold water. The solids were subsequently recovered through filtration and were recrystallized from ethanol, yielding 93% (4.74 g) of yellow crystals, m.p. 264.5-266.1 °C. ^1^H NMR (600 MHz, DMSO-d_6_) 2.47 (*s*, 1H, SH) 7.26 (*d*, 1H, *J_o_* = 8.8 Hz, H-7), 7.85 (*d*, 1H, *J_m_* = 2.3 Hz, H-4), 8.04 (*dd*, 1H, *J_m_* = 2.4, *J_o_* = 8.6 Hz, H-6), ppm. ^13^C NMR (150 MHz, DMSO-d_6_) 105.1 (C-7), 109.7 (C-4), 119.4 (C-6) 132.8 (C-7a), 138.0 (C-3a), 143.0 (C-5), 172.3 (C-2) ppm; MS: *m/z* (% rel. int.) 195 (M^+^, 100%), 149 (M-46, 38%).

#### 3.1.3. 5-Nitro-2-(propylthio)-1*H*-benzo[d]imidazole (**3**)

Compound **3** was prepared following the procedure outlined by Soria-Arteche [32]. Initially, a solution of **2** in acetonitrile was prepared, to which K_2_CO_3_ (1.41 g, 10.2 mmol, 2 equiv) diluted in water was added. The reaction mixture was stirred for 15 min at room temperature. Subsequently, a mixture of the propyl iodide diluted in acetonitrile was added dropwise. The resulting mixture was stirred for 12 h at 2–3 °C. Thereafter, the solvent was removed using a high-vacuum system, and the residue was suspended in cold water. The solid components were recovered through filtration, and the product was recrystallized from ethanol, giving a yellow solid, yield 90% (1.09 g), m.p. 91.9–93.7 °C. ^1^H NMR (600 MHz, DMSO-d_6_) 1.07 (*t*, 3H, *J* = 7.4 Hz, H-3′) 1.80 (sext, 2H, *J* = 7.3 Hz, H-2′), 3.29 (*t*, 2H, *J* = 7.2 Hz, H-1′), 7.50 (*d*, 1H, *J_o_* = 8.8 Hz, H-7), 8.09 (*dd*, 1H, *J_m_* = 2.1, *J_o_* = 8.8 Hz, H-6), 8.30 (s, 1H, H-4), ppm. ^13^C NMR (150 MHz, DMSO-d_6_) 12.0 (C-3′), 22.7 (C-2′), 33.2 (C-1′) 109.8 (C-7), 112.6 (C-4), 117.5 (C-6), 143.0 (C-5), 157.1 (C-2) ppm; MS: *m/z* (% rel. int.) 237 (M^+^, 18%), 195 (M-42, 100%).

#### 3.1.4. 2-(Propylthio)-1*H*-benzo[d]imidazol-5-amine (**4**)

To a mixture of **3** (1 g, 4.2 mmol) in ethanol, Sn^0^ (3 g, 25.2 mmol, 6 equiv) and concentrated HCl (7 equiv, 721 mL) were added; the reaction mixture was stirred for 15 min at room temperature. After, the mixture was stirred at reflux for 12 h. At the end of the reaction, it was neutralized to pH 7 with a saturated NaHCO_3_ solution; it was filtrated and the solvent was removed with a high-vacuum system, and the amine liquid product was used immediately for the next reaction, due to its chemical instability.

#### 3.1.5. Methyl (2-(propylthio)-1*H*-benzo[d]imidazol-5-yl)carbamate (**RetroABZ**, **1**)

To a solution of amine **4** (1 g, 4.8 mmol) in CH_2_Cl_2_, a mixture of the corresponding methyl chloroformate (0.5 g, 4.8 mmol, 1 equiv) diluted in CH_2_Cl_2_ was added dropwise. This mixture was stirred at 2–3 °C for 8 h. At the end of the reaction, it was neutralized to pH 7 with a NaHCO_3_ solution, the solvent was removed with a high-vacuum system, and the residue was suspended in cold water. The solids were recovered with filtration, and purified with a chromatography column, giving a brown solid with m.p. 179.5–183.5 °C, yield 72% (0.92 g). ^1^H NMR (600 MHz, DMSO-d_6_) 0.99 (*t*, 3H, *J* = 7.3 Hz, H-3′) 1.71 (sext, 2H, *J* = 7.3 Hz, H-2′), 3.47 (*t*, 2H, *J* = 7.2 Hz, H-1′), 7.41 (*dd*, 1H, *J_m_ = 2.0, J_o_* = 8.8, H-6), 7.56 (*d*, 1H, *J_o_* = 8.8 Hz, H-7), 7.94 (*s*, 1H, H-4), 9.98 (*s*, 1H, carbamate) ppm; ^13^C NMR (150 MHz, DMSO-d_6_) 13.2 (C-3′), 23.0 (C-2′), 34.5 (C-1′), 52.3 (C-5′), 101.8 (C-4), 113.8 (C-7), 116.9 (C-6), 128.8 (C-5), 133.6 (C-7a), 137.3 (C-3a), 150.2 (C-2), 154.5 (C=O) ppm; MS: *m/z* (% rel. int.) 265 (M^+^, 16%), 223 (M-42, 20%), 191 (M-74, 100%).

### 3.2. Biological Assays

#### 3.2.1. In Vitro Giardicidal Assay

*G. lamblia* strain WB was cultured in a TYI-S-33 medium (Manassas, VA, USA) supplemented with 10% calf serum and bovine bile. In vitro susceptibility tests were conducted using 4 × 10^4^ trophozoites of *G. lamblia*. These trophozoites were incubated for 48 h at 37 °C with incremental concentrations of both **RetroABZ** and albendazole, using DMSO (0.05%) as a cosolvent. Additionally, a control group was incubated solely in a culture medium containing DMSO. Following the incubation period, the trophozoites were washed and subcultured in a fresh medium without drugs for an additional 48 h. Subsequently, the trophozoites count was performed, and the median inhibitory concentration (IC_50_) was calculated with a Probit analysis. All experimental procedures were replicated in triplicate [19,33].

#### 3.2.2. In Vitro Cytotoxic Assay

The cytotoxic assay of **RetroABZ** was carried out in Huh-7 (human hepatocellular carcinoma cell line) cells acquired from ATCC (American Type Culture Collection, Manassas, VA, USA). We also included an immortalized human epidermal keratinocyte line (HaCaT) as a control of non-cancerous cells [34]. The cells were cultured in a DMEM medium (Invitrogen, Thermo Fisher Scientific, Inc., Waltham, MA, USA), supplemented with 10% fetal bovine serum (SFB, Invitrogen) and 2 mM glutamine. All cultures were maintained under standard culture conditions (37 °C and 5% CO_2_). For the cytotoxic assessment in a 96-well plate, 5000 cells per well were planted. A stock solution of **RetroABZ** was solubilized in DMSO, and a concentration/response curve was generated using concentrations of 100, 10, 1, 0.1, and 0.01 μg/mL for Huh-7, while for Hacat (200, 20, 2, 0.2, and 0.02) and negative control 0.05% DMSO in a culture medium, the cells were then incubated for 48 h. The cellular viability was next calculated utilizing the CellTiter 96^®^ AQueous One Solution Cell Proliferation Assay kit (Promega, Madison, WI, USA), following the manufacturer’s instructions [35]. The experiments were carried out in triplicate in separate experiments. The Prism 8.0 statistical tool (Graphpad Software Inc., La Jolla, CA, USA) was used to examine the data, and a regression analysis was used to obtain the IC_50_.

#### 3.2.3. In Vivo Antigiardial CD-1 Mouse Model

The in vivo antigiardial activity of the compounds was assessed following a previously described phenotypic drug screening method [20,36]. Female CD-1 mice (16–24 g) were obtained from the animal house facility at Universidad Autónoma Metropolitana, Xochimilco, and were maintained under standard laboratory conditions. The experimental protocols were reviewed and approved by the Animal Care and Use Committee, ensuring compliance with the guidelines for the care and use of laboratory animals. The giardicidal effect of **RetroABZ** was evaluated on mice infected with trophozoites of the *G. lamblia* WB strain. Mice were orally treated with a mebendazole solution (10 mg/mouse/day) for 3 consecutive days to ensure their freedom from any potential protozoan infections. One week after treatment, three consecutive fecal examinations were conducted to confirm the absence of protozoan infections before proceeding with experimental infection. Mice (*n* = 6 per group) were intragastrically infected with 1 × 10^6^ trophozoites in 300 µL of a TYI-S-33 medium (Manassas, VA, USA) using a silastic tube (0.0025 in. outside diameter) attached to a 1 mL syringe. Six days after infection, mice were treated intragastrically with 300 µL of **RetroABZ** dissolved in 1 mL of a 2% DMSO solution in water. Albendazole was included as a positive control and a control group received the vehicle alone (2% DMSO solution). Five single doses (0.018, 0.037, 0.075, 0.15, and 0.3 mg/kg) of both compounds were evaluated. Forty-eight hours after the treatment (8 days post-infection), the mice were euthanized, and the entire small intestine was removed, opened longitudinally, and placed in 10 mL of a 10% PAF fixer for a minimum of 2 h. Tubes were then vortexed to ensure complete detachment of the trophozoites, and the number of organisms present was calculated for both the treated and control groups. Five single doses of selected compounds were tested, with albendazole used as a positive control [20].

#### 3.2.4. Statistical Analysis

A data analysis was performed utilizing a Probit analysis. The percentage of surviving trophozoites was calculated by comparing their growth with the control group. To determine the dose required to kill 50% of the trophozoites (ED_50_), along with the 95% confidence limits, a Probit plot against the logarithm of the drug concentration was generated.

### 3.3. Determination of Thermodynamic Solubility

The aqueous solubility of **RetroABZ** was assessed using the shake-flask saturation method, as described in previous studies [37]. To prepare saturated solutions of the compound, an excess amount of **RetroABZ** and albendazole (6 mg) was stirred into 6 mL of bidistilled water for 48 h at a constant temperature of 37 ± 2 °C. Subsequently, the solution was filtered through a 0.2 μm filter (Phenex-NY 4mm, Torrance, CA, USA). The aqueous solubility of the compound was then determined using an HPLC method. Experimentally, the compound **RetroABZ** was analyzed with high-performance liquid chromatography (HPLC PerkinElmer 200, Waltham, Massachusetts). The HPLC analysis was performed using an ultraviolet detector (UV/VIS 200). The analite was monitored at a wavelength of 254 nm. Chromatographic separation was performed using a Phenomenex Luna C18 (4.6 × 150 mm, 5 µm, Torrance, CA, USA). The mobile phase utilized in the study consisted of a mixture of methanol and water in a ratio of 60:40 (*v*/*v*). The elution from the stationary phase was conducted at a constant flow rate of 1 mL/min (isocratic mode). Aliquots of 20 µL were injected into the HPLC system and the total run time for each injection was set at 10 min; the analysis was performed for triplicate. Albendazole was assessed using the method reported by Jung et al. [38]. Albendazole was analyzed with high-performance liquid chromatography, monitored at a wavelength of 254 nm. Chromatographic separation was performed using a Phenomenex Luna C18 (4.6 × 150 mm, 5 µm). The mobile phase utilized was a mixture of methanol and a 0.05 M phosphate buffer (pH 5.7), 70:30 (*v*:*v*). The flow rate was kept constant at 1 mL/min. To prepare the saturated solution of the compound (1 mg/mL), it was stirred for 48 h at a constant temperature of 37 ± 2 °C. Subsequently, the solution was filtered through a 0.2 µm filter (Phenex-NY 4 mm). Aliquots of 20 µL were injected into the HPLC system, and the analysis was performed for triplicate.

### 3.4. In Silico Studies

#### 3.4.1. Ligand Preparation

The tridimensional structures of albendazole and **RetroABZ** were constructed and processed using the OpenBabel toolbox [39]. The structures underwent additional parametrization within the PlayMolecule web server [40] using General Amber force field 2 (GAFF2) with AM1-BCC atomic charges, and employing ANI-2x for dihedral angle fixing [40,41,42]. For the dihedral fitting, we used the DFT (density-functional theory) functional wB97X-D with the quantum mechanics basis 6–311++G**. The resultant Amber topologies produced with the platform were translated into GROMACS files (.gro and .itp) using the Antechamber Python Parser Interface (ACPYPE) [43]. Once again, partial charges were set with AM1–BCC and GAFF2 atom types were used.

#### 3.4.2. Homology Modelling

Currently, there is a lack of experimentally determined crystallographic structures for *G. lamblia* α- and β-tubulin. However, alternative approaches, such as homology modeling, have been utilized in previous studies as an alternative approach [26]. In our research, we embarked on the task of creating a model for *G. lamblia* α- and β-tubulin using MOE 2020.0901 software. Specifically, we focused on amino acid residues 1–438 of the α-tubulin and 1–428 of the β-tubulin sequence of *G. lamblia*. To accomplish this, we carefully selected a suitable template protein from the Protein Data Bank (PDB) that exhibited homologous characteristics with our target protein. A suitable template was identified in *Gallus gallus* tubulins (PDB ID: 7DBC) for our modeling purposes [44]. The selection of this particular template was based on its association with a ligand–receptor complex involving the carbamate-type compound parbendazole, which played a crucial role in our decision-making process. An interesting observation is the molecular resemblance between the cocrystallized ligand and **RetroABZ**, implying a potential similarity in their binding modes. The generated model was assessed using the QMEAN score through the web interface (https://swissmodel.expasy.org/qmean/ accessed on 12 November 2022) [45] and the overall geometry quality of the model was evaluated using the Ramachandran plot constructed with MOE 2020.0901 (Montreal, QC, Canada).

#### 3.4.3. Molecular Docking

Docking calculations were performed using Gnina 1.0 [46]. Except for the amide group, all torsions of the ligands were allowed to rotate. For the protein, Gasteiger charges were assigned, and non-polar hydrogen atoms were merged. The search box was centered on the parbendazole binding site of the *G. lamblia* α/β-tubulin model, with dimensions of 30 Å on each side. An exhaustiveness value of 100 was utilized for the search and the convolutional neural network (CNN) score was employed to retrieve the most reliable pose of each ligand. The molecular docking protocol was validated by redocking the parbendazole into the binding site on the β-tubulin model. The root-mean-square deviation (RMSD) between the reference ligand and the docked structures was less than 2.0 Å. This value indicates that the parameters for docking simulations are good in reproducing the pose in the protein.

#### 3.4.4. Molecular Dynamics

Molecular dynamics (MD) simulations were conducted to assess the stability of protein–ligand complexes. For this purpose, three independent simulations of albendazole and **RetroABZ** (MD1 to MD3) bound to the *G. lamblia* α/β-tubulin heterodimer were carried out, each spanning 100 ns. All the simulations were performed using the GROMACS 2022.5 [47] software package and the AMBER14SB force field [48]. The systems were solvated in cubic boxes using the TIP3P water model and were neutralized by introducing sodium and chloride ions to achieve a concentration of 0.15 M. Topologies of GTP and GDP molecules were taken from Meagher et al.’s [49] study. The simulations included energy minimization using the steepest-descent algorithm and equilibration for 1 ns under both NVT and NPT ensembles in each system. The temperature was held at 300 K through the V-rescale coupling thermostat [50], while the Parrinello–Rahman barostat [51] maintained isotropic pressure at 1.0 bar. The Linear Constraint Solver (LINCS) algorithm was employed to apply holonomic constraints to hydrogen atom bonds. A cutoff radius of 1.2 nm was set for van der Waals and short-range electrostatic interactions, whereas the Particle Mesh Ewald (PME) method was used for approximating long-range electrostatic calculations. The MD analysis was conducted using GROMACS built-in tools. This included calculating the backbone root-mean-square deviation (RMSD), ligand RMSD after fitting it to the tubulin backbone using least squares, hydrogen bonding interaction fraction (IF_HB_), and clustering analysis based on ligand RMSD using a 0.15 nm cutoff with the gromos method. The hydrophobic interaction fraction (IF_HI_) was computed using the Protein-Ligand Interaction Profiler (PLIP) [52]. Finally, the binding free energy (∆G_bind_) was determined using the Molecular-Mechanics–Poisson–Boltzmann Surface Area (MM/PBSA) approach with the gmx_MMPBSA [53] tool. We employed the AMBER14SB force field, modified GB model 1 (GB-OBC1) for solvent polarization calculations, and the interaction entropy (IE) [54] method to compute the entropic component. The intermolecular interactions, clustering analysis, and binding free energy calculations were computed from the final 50 ns of the three MD simulations.

## 4. Conclusions

In this study, we present the design and synthesis of a novel regioisomer of albendazole, called **RetroABZ**. This molecule demonstrated highly potent in vitro activity against *G. lamblia*, displaying remarkable efficacy in the nanomolar range. Notably, **RetroABZ** exhibited oral activity in a mouse model of giardiasis, surpassing the reference drug, albendazole, by an impressive 11-fold difference in terms of efficacy. Furthermore, the compound exhibited very low cytotoxicity and displayed a high selectivity index against *G. lamblia*. A notable observation is that the strategic exchange of side chains in the molecular design not only maintained but also augmented the compound’s activity, along with an exceptional 23-fold increase in solubility compared to the reference drug. This enhancement in solubility is particularly noteworthy, as low water solubility is a known limitation of benzimidazole carbamate compounds. With its compelling in vitro and in vivo antiparasitic properties and improved solubility, the compound **RetroABZ** opens new avenues for the development of bioactive molecules targeting systemic parasites. On the other hand, the limitations of this study lie in the fact that the findings are in the early preclinical stage. Upon identifying a lead candidate (**RetroABZ**), the customary progression in preclinical development involves several key endeavors: synthesizing the active pharmaceutical ingredient, preformulation studies, development and validation of analytical and bioanalytical methods, as well as assessments of metabolism, pharmacokinetics, and toxicology. Furthermore, it would be imperative to evaluate the compound on *Giardia* strains that have demonstrated resistance to benzimidazole carbamates. Ongoing efforts are directed toward further optimizing this compound to gain deeper insights into its structure–activity relationships.

## Figures and Tables

**Table 1:** Physicochemical properties, in vitro bioactivity, and cytotoxicity of **RetroABZ** and reference compound albendazole.

Compound		IC_50_ (μM)	CC_50_ (μM)		Selectivity Index	
	Mp (°C)	*G. lamblia*	Huh-7	HaCaT	CC_50_/IC_50_	
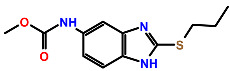 **RetroABZ**	179.5–183.5	0.083 ± 0.013	312.8 ± 22.2	587.94 ± 18.47	**Huh-7**	**HaCaT**
					3768	7083
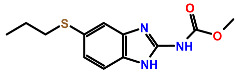 Albendazole	209.1–210.5	0.196 ± 0.011	79.71 ± 1.0	118.2 ± 1.0	407	603

**Table 2:** In vivo assay (ED_50_) of RetroABZ, albendazole, and metronidazole against *G. lamblia* (WB strain) in experimentally infected CD-1 mice.

Compound	ED_50_ (μg/kg)	ED_50_ (nmol/kg)
**RetroABZ**	5	18.8
Albendazole	55	207.3
Metronidazole	194	1133.5

**Table 3:** Solubility of **RetroABZ** and albendazole in water.

Compound	Conditions	Solubility (μg/mL)
**RetroABZ**	37 ± 2 °C	245.74 ± 4.07
Albendazole	37 ± 2 °C	10.55 ± 0.70

**Table 4:** Pharmacokinetic predictive values calculated with ADMETLab 2.0 for compounds **RetroABZ** and albendazole.

Model		Compounds	
		RetroABZ 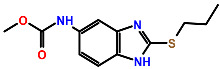	Albendazole 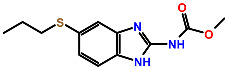
A	Gastrointestinal absorption	(+++)	(+)
	Blood–brain barrier permeant	(++)	(−−)
D	Plasma protein binding	96%	80%
	Volume distribution	0.68 L/kg	0.69 L/kg
M	CYP3A4 substrate	(−−) Low	(−−) Low
	CYP2D6 substrate	(++) Medium	(++) Medium
E	Clearance	6.0 mL/min/Kg	6.9 mL/min/Kg
	Half-life (T _½_)	>3 h	>3 h
T	hERG blockers	(−−−)	(−−)
	Rat oral acute toxicity	(−−−)	(−−)

The prediction probability values are transformed into six symbols: 0–0.1 (−−−), 0.1–0.3 (−−), 0.3–0.5 (−), 0.5–0.7 (+), 0.7–0.9 (++), and 0.9–1.0 (+++).

**Table 5:** Consensus analysis of **RetroABZ** versus albendazole.

	RetroABZ	Albendazole	Winner
In vitro IC_50_ *G. lamblia* (μM)	0.083 ± 0.013	0.189 ± 0.011	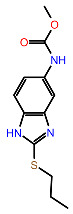 **RetroABZ**
In vivo ED_50_ *G. lamblia* (μg/kg)	5	55	
SI CC_50_ HaCaT/IC_50_ *G. lamblia*	7083 □	603	
SI CC_50_ Huh-7/IC_50_ *G. lamblia*	3768	407	
Solubility ≥ 60 μg/mL	245.74 ± 4.07	10.55 ± 0.70 □	
In silico affinity ΔG_bind_ (kcal/mol)	−14.09 ± 3.65	−11.57 ± 3.76	
In silico pharmacokinetic profile	□	□	
ClogP (2–3)	2.91	2.08	

To visualize the pharmacological consensus analysis, we used a color code indicating chances that the compound has drug-like properties, as follows: green (very satisfactory) and red (unsatisfactory).

## Data Availability

Not applicable.

## Supplementary Materials

The following supporting information can be downloaded at: https://www.mdpi.com/article/10.3390/ijms241914949/s1.
