# Supplementary material for: Enhancing Giardicidal Activity and Aqueous Solubility through the Development of “RetroABZ”, a Regioisomer of Albendazole: In Vitro, In Vivo, and In Silico Studies
Source: Int J Mol Sci. 2023 Oct 6;24(19):14949. doi: 10.3390/ijms241914949 (PMC10573946; doi:10.3390/ijms241914949)
Supplement: Supplementary file 1 [file ijms-24-14949-s001.zip › ijms-2645490-supplementary.pdf]

Supplementary Material

Enhancing Giardicidal Activity and Aqueous Solubility through the Development of “RetroABZ”, a Regioisomer of Albendazole: In Vitro, In Vivo, and In Silico Studies †

Carlos Martínez-Conde [1], Blanca Colín-Lozano [1], Abraham Gutiérrez-Hernández [1], Emanuel Hernández-Núñez [2], Lilián Yépez-Mulia [3], Luis Fernando Colorado-Pablo [4], Rodrigo Aguayo-Ortiz [4], Jaime Escalante [5],
Julio C. Rivera-Leyva [1], Jessica Nayelli Sánchez-Carranza [1], Elizabeth Barbosa-Cabrera [6] and Gabriel Navarrete-Vazquez [1,*]

[1] Facultad de Farmacia, Universidad Autónoma del Estado de Morelos, Cuernavaca 62209, Morelos, Mexico; mcc_ff@uaem.mx (C.M.-C.); clbi_ff@uaem.mx (B.C.-L.); ghaa_ff@uaem.mx (A.G.-H.); julio.rivera@uaem.mx (J.C.R.-L.); jessica.sanchez@uaem.mx (J.N.S.-C.)

[2] Departamento de Recursos del Mar, Centro de Investigación y de Estudios Avanzados, IPN, Unidad Mérida, Merida 97310, Yucatán, Mexico; emanuel.hernandez@cinvestav.mx

[3] Unidad de Investigación Medica en Enfermedades Infecciosas y Parasitarias, Unidad Médica de Alta Especialidad-Hospital de Pediatría, Centro Médico Nacional Siglo XXI, Instituto Mexicano del Seguro Social, Mexico City 06720, Mexico; lilianyepez@yahoo.com

[4] Departamento de Farmacia, Facultad de Química, Universidad Nacional Autónoma de Mexico, Mexico City 04510, Mexico; fernando1303@comunidad.unam.mx (L.F.C.-P.); rodaguayo@comunidad.unam.mx (R.A.-O.)

[5] Centro de Investigaciones Químicas-IICBA, Universidad Autónoma del Estado de Morelos, Av. Universidad 1001, Cuernavaca 62209, Morelos, Mexico; jaime@uaem.mx

[6] Sección de Estudios de Posgrado e Investigación, Escuela Superior de Medicina, IPN, Mexico City 11340, Mexico; rebc78@yahoo.com.mx

* Correspondence: gabriel_navarrete@uaem.mx; Tel.: +52-777-329-7089 (ext. 2322)

† Taking in part of the Ph.D. thesis of C. Martinez-Conde.

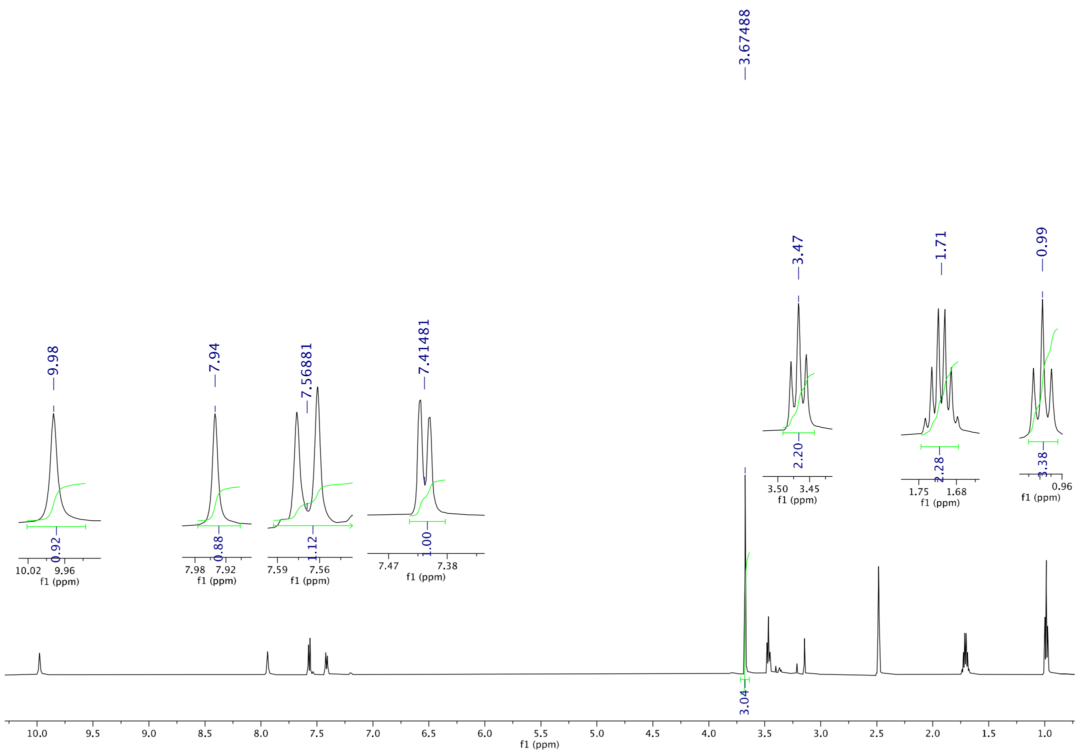

^{1}H NMR: *Methyl (2-(propylthio)-1H-benzo[d]imidazol-5-yl)carbamate* (**RetroABZ, 1**)

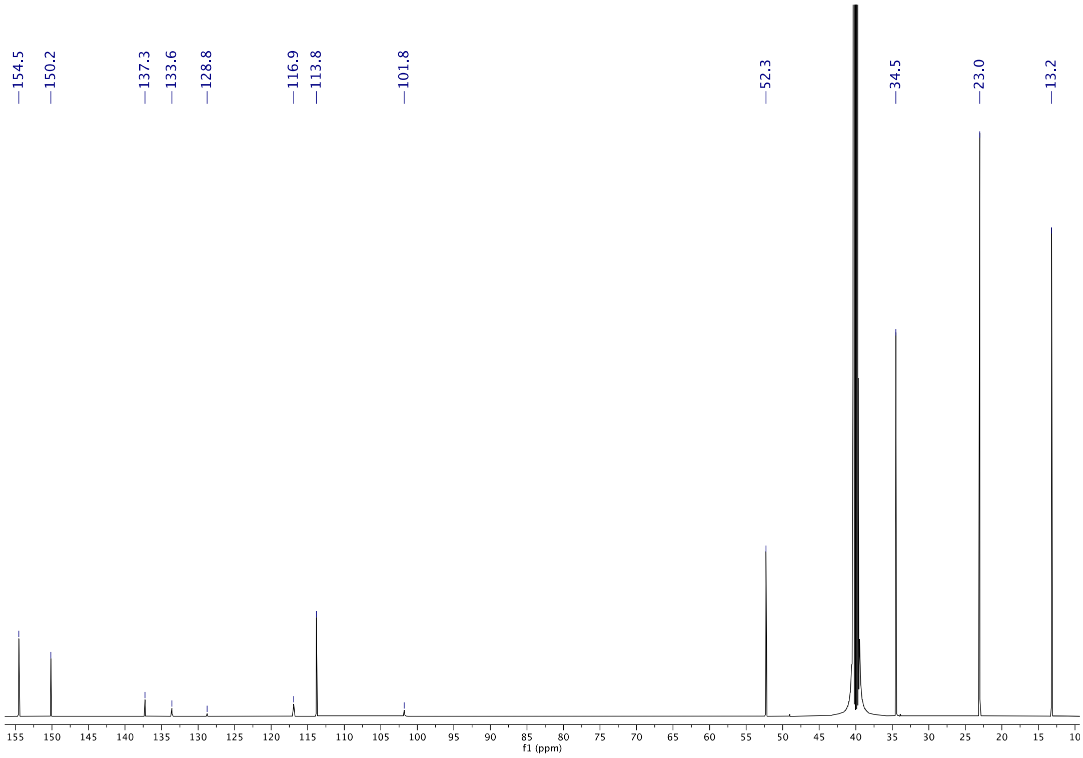

^{13}C NMR: *Methyl (2-(propylthio)-1H-benzo[d]imidazol-5-yl)carbamate* (**RetroABZ, 1**)

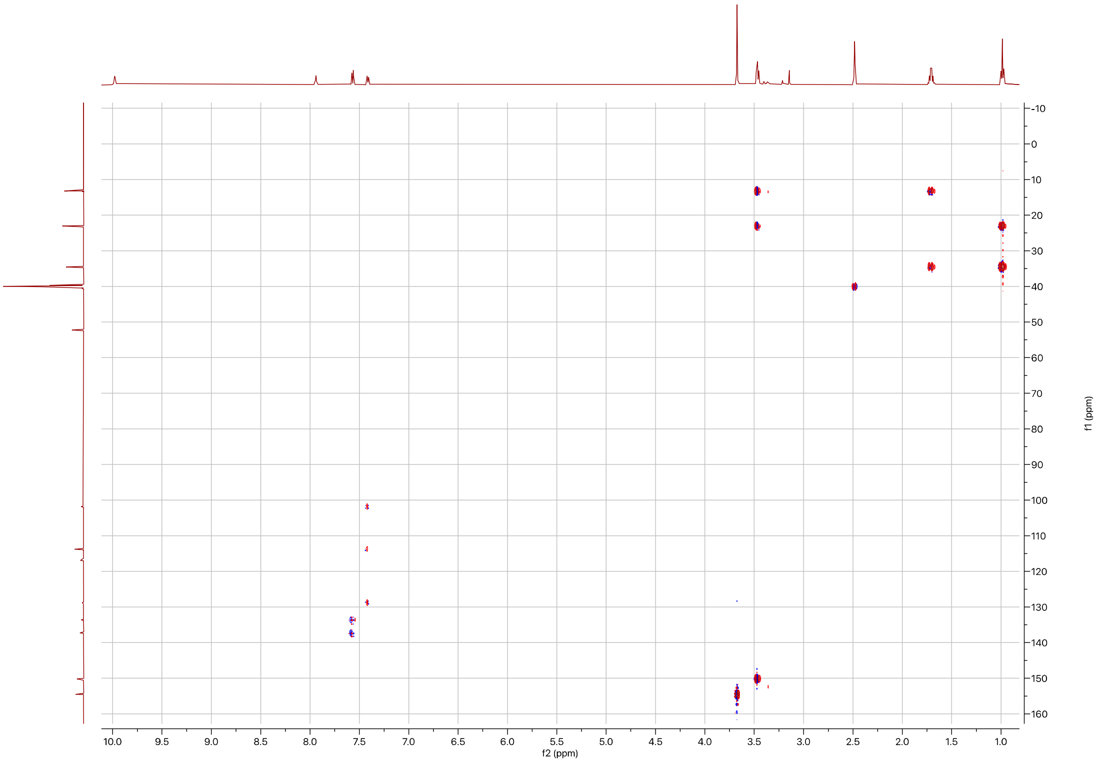

HMBC, *Methyl (2-(propylthio)-1H-benzo[d]imidazol-5-yl)carbamate* (**RetroABZ, 1**)

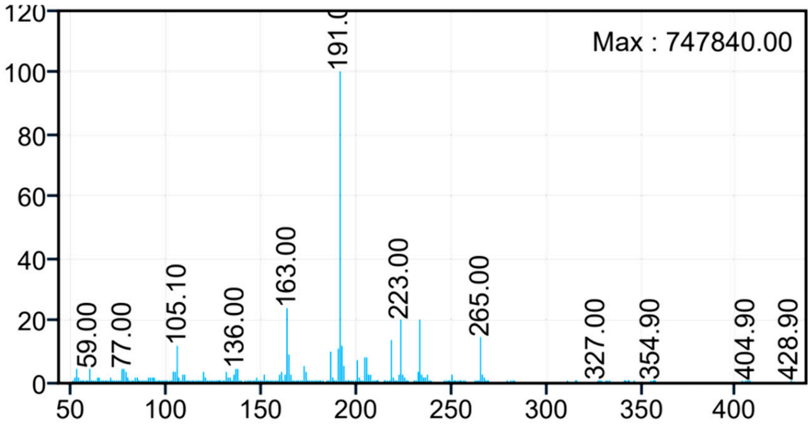

MS: *m/z* (% rel. Int), *Methyl (2-(propylthio)-1H-benzo[d]imidazol-5-yl)carbamate* (**RetroABZ, 1**)

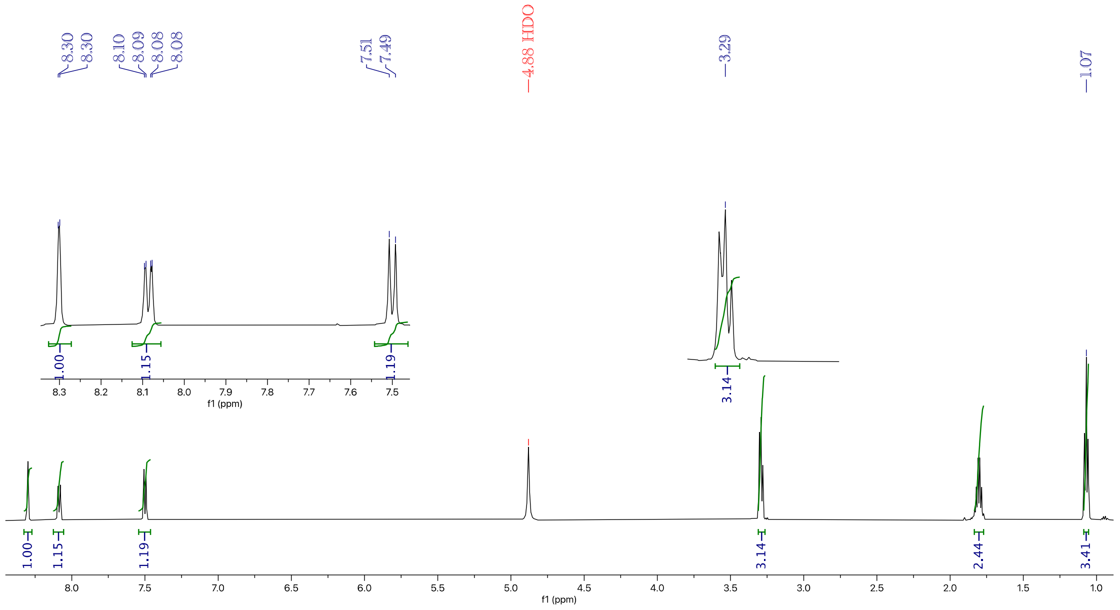

^{1}H NMR, *5-Nitro-2-(propylthio)-1H-benzo[d]imidazole*

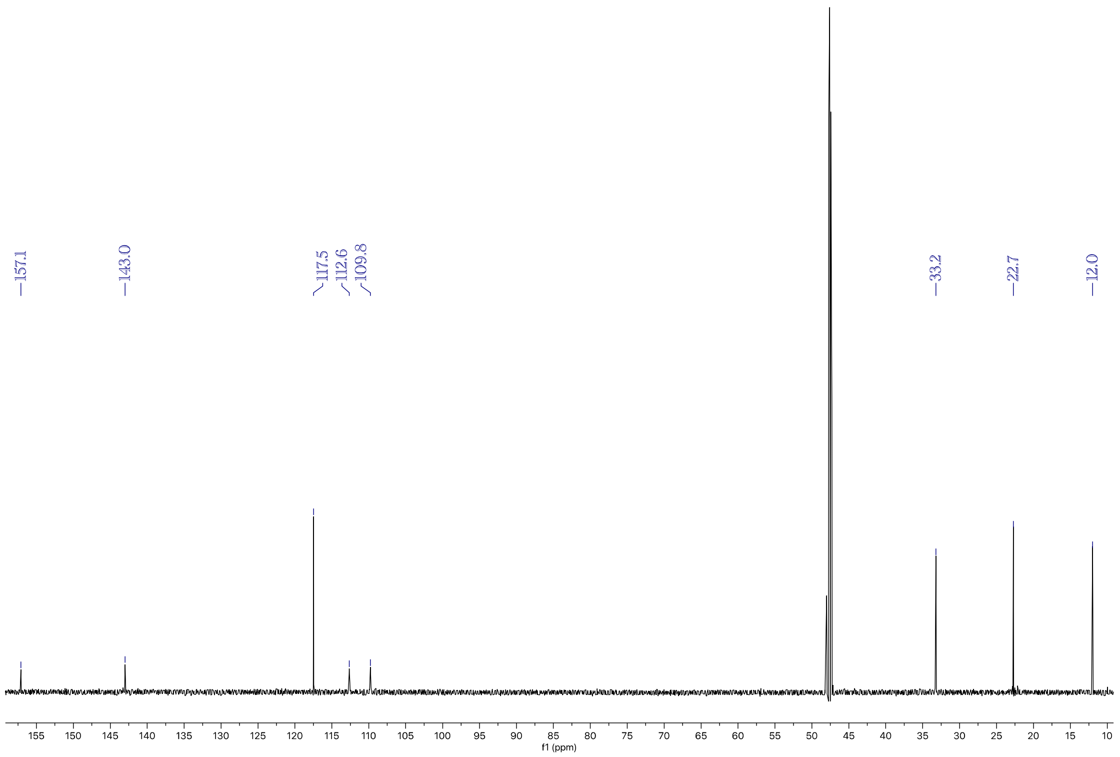

^{13}C NMR, *5-Nitro-2-(propylthio)-1H-benzo[d]imidazole*

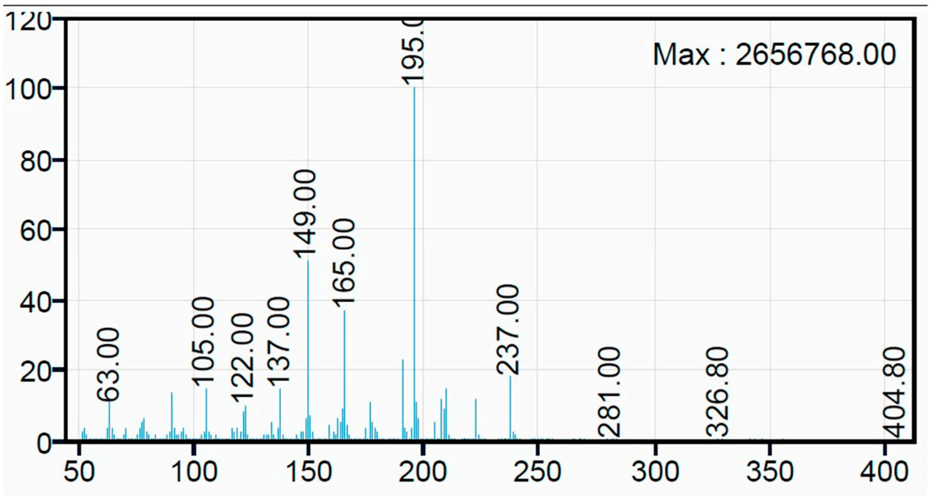

MS: *m/z* (% rel. Int), *5-Nitro-2-(propylthio)-1H-benzo[d]imidazole*

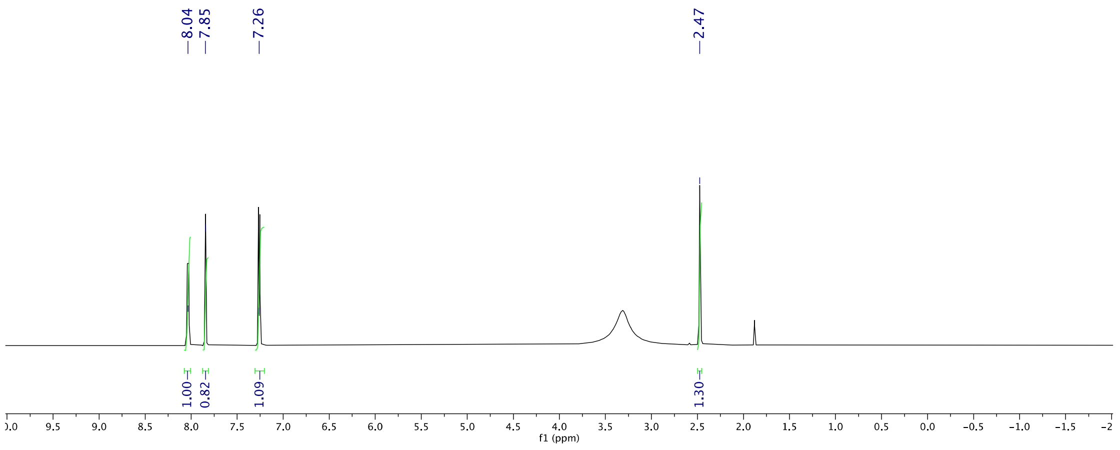

^{1}H NMR, *5-Nitro-1H-benzo[d]imidazole-2-thiol* (***2***)

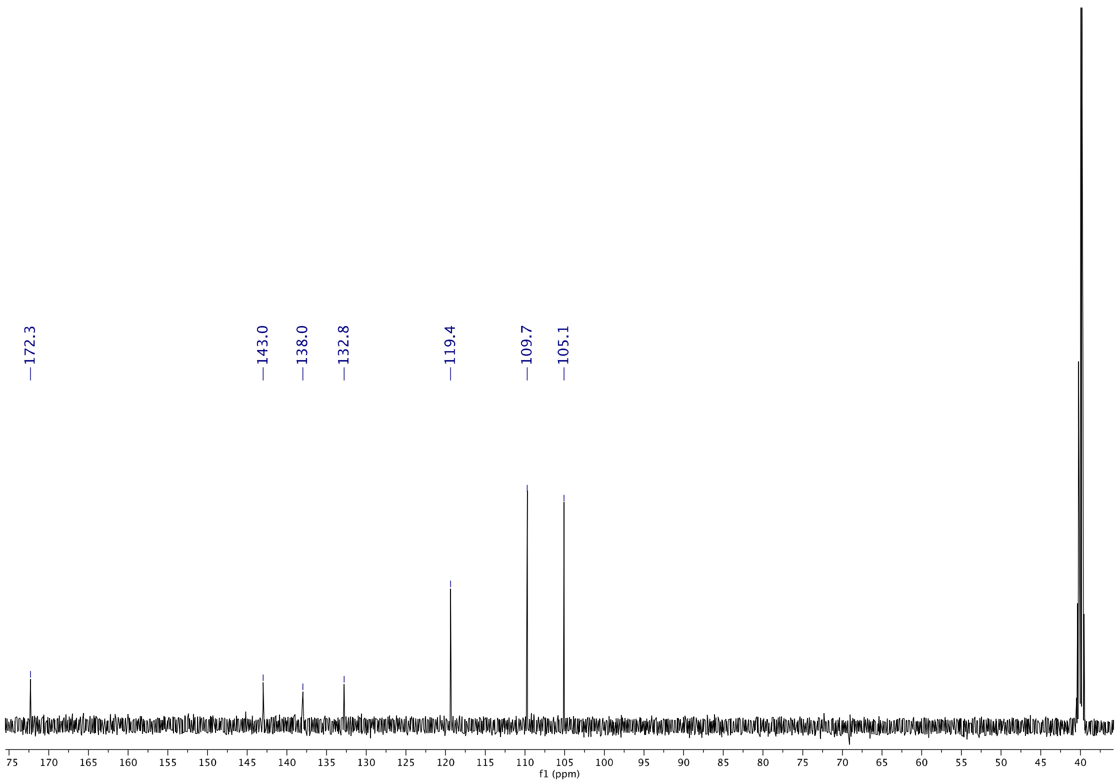

^{13}C NMR, *5-Nitro-1H-benzo[d]imidazole-2-thiol* (***2***)
